# ML-Based Edge Node for Monitoring Peoples’ Frailty Status

**DOI:** 10.3390/s24134386

**Published:** 2024-07-05

**Authors:** Antonio Nocera, Linda Senigagliesi, Gianluca Ciattaglia, Michela Raimondi, Ennio Gambi

**Affiliations:** Department of Information Engineering, Università Politecnica delle Marche, via Brecce Bianche 12, 60131 Ancona, Italy; a.nocera@pm.univpm.it (A.N.); g.ciattaglia@staff.univpm.it (G.C.); m.raimondi@staff.univpm.it (M.R.); e.gambi@staff.univpm.it (E.G.)

**Keywords:** deep learning, edge computing, frailty, machine learning, Raspberry Pi4, RGB, video

## Abstract

The development of contactless methods to assess the degree of personal hygiene in elderly people is crucial for detecting frailty and providing early intervention to prevent complete loss of autonomy, cognitive impairment, and hospitalisation. The unobtrusive nature of the technology is essential in the context of maintaining good quality of life. The use of cameras and edge computing with sensors provides a way of monitoring subjects without interrupting their normal routines, and has the advantages of local data processing and improved privacy. This work describes the development an intelligent system that takes the RGB frames of a video as input to classify the occurrence of brushing teeth, washing hands, and fixing hair. No action activity is considered. The RGB frames are first processed by two Mediapipe algorithms to extract body keypoints related to the pose and hands, which represent the features to be classified. The optimal feature extractor results from the most complex Mediapipe pose estimator combined with the most complex hand keypoint regressor, which achieves the best performance even when operating at one frame per second. The final classifier is a Light Gradient Boosting Machine classifier that achieves more than 94% weighted F1-score under conditions of one frame per second and observation times of seven seconds or more. When the observation window is enlarged to ten seconds, the F1-scores for each class oscillate between 94.66% and 96.35%.

## 1. Introduction

Autonomy, defined as the ability to be independent in the performance of activities of daily living, is one of the parameters identified to assess of the state of frailty in the elderly population [1,2,3,4,5]. Activities of daily living, such as those related to hygiene, play an important role in the maintenance of physical and mental wellbeing. Oral health has been associated with systemic diseases [6] and contributes to the general health conditions of people [7]. Washing hands is one of the suggested practices to prevent the diffusion of disease, as demonstrated during the COVID-19 pandemic [8]. Thus, elderly people must be supported and encouraged to perform personal hygiene, and a loss of initiative or ability to perform these actions should be noted. Most people have been autonomous since childhood in providing personal hygiene, and as such may find it difficult to accept the presence of caregivers during this task. Nevertheless, early signs of lack of self-care can be an indication of the so-called “state of frailty”, which precedes loss of autonomy. Identifying frailty and demonstrating proper intervention is crucial to prevent worse repercussions on the individual’s quality of life. In addition, this can decrease the burden on families and healthcare systems.

The development of methods for automatically recognising the absence of hygiene-related activities without disrupting normal living could aid in the prevention of frailty-related risks. Tools for determining adequate oral hygiene have been proposed, such as toothbrushes incorporating localised accelerometers and machine learning (ML) or deep learning (DL) algorithms to check for proper brushing technique [9,10]. Proper hand washing protocol can be automatically verified by environmental sensors such as video cameras or radars. The provided data can be analysed and classified in terms of the desired actions by ML or DL [11,12,13,14,15]. Papers dealing with hygiene-related activity often focus on determining whether the task is performed correctly. The evaluation of hand washing usually consists of an automatic check of the protocol proposed by the World Health Organisation [12,13,14,15]. The system uses Mediapipe algorithms as feature extractors [11,13] or works directly on RGB images [12,14,15], achieving high performance with ML and/or DL; however, neither approach was tested for use on platforms with limited computational resources. Similarly, tooth brushing activity is often divided into other small subtasks that need to be classified for a complete cleaning protocol. The steps in the tooth brushing protocol are often classified using small processors that work with embedded sensors recording the acceleration of the toothbrush [9,10].

A crucial aspect when implementing this monitoring technology is its integration with the environment. In recent years, the Internet of Things (IoT) has become one of the most relevant technologies for achieving this purpose. Remarkable progression in sensor-based methods has led to the rapid evolution of IoT applications for the development of real-time monitoring systems. In modern healthcare applications, the use of IoT brings physicians and patients together for automated and intelligent monitoring of the daily activities of elderly patients. The advent of the IoT has led to the development of home security systems, often using Raspberry Pi as the development board [16,17,18]. These systems consist of algorithms for detecting and authenticating a person entering at the home using RGB images. Usually, the algorithms involve the use of simple feature extractors, such as locally binary patterns, and the face is authenticated by comparing histograms. When the task becomes the classification of different activities, the complexity of the algorithms employed increases with the use of ML and DL models; however, the complexity of the algorithms is limited by the computational capabilities of the platform on which it operates. The Raspberry Pi has commonly been used as the main computational resource in papers regarding gesture recognition [19], sign language recognition [20], and fall detection [21]. In conditions of low computing power, such as in the case of mobile platforms, MediaPipe [22] is often used as a framework for extracting pose, face and/or hand keypoints from RGB images. These features can then be classified according to the user’s objective. The advantage of using the MediaPipe framework lies in its low inference time. MediaPipe Hands, a DL algorithm developed by Google for fast inference on mobile platforms, has been used to simplify the gesture and sign language recognition process by providing the correct hand joint coordinates [19,20]. These coordinates are then sent to a classifier for the final classification task. On the other hand, MediaPipe Pose, another DL algorithm from the same library, was used in [21] to extract the torso and arm joint coordinates, then a random forest algorithm was used to predict falls. Table 1 provides a comparison between existing state-of-the-art works and our proposal, highlighting differences and the novelty of our approach. To the best of our knowledge, there are no existing works providing for continuous-time monitoring of self-hygiene and related activities with an edge implementation on a Raspberry Pi4B.

### Contributions and Paper Structure

This paper proposes a system for classifying and monitoring whether an activity is performed. As mentioned earlier, the lack of self-care is an indication of frailty; therefore, this paper aims to classify three main classes of hand washing, face washing, and tooth brushing, to which we add a fourth ‘no action’ class. Our aim is not to assess the quality of the cleaning procedure, but rather to understand whether the procedure is performed at all. This system could help to detect early signs of loss of self-care, helping to make prompt interventions to improve the subject’s quality of life. Senigagliesi et al. [24] previously proposed an Long Short-TermMemory (LSTM)-based system to classify the same four actions. Here, however, the implementation of an edge node is proposed using a Raspberry Pi 4B with Pi camera. To this end, a simpler ML alternative is chosen. A public dataset, Kinetics-700 [25], containing the selected three hygiene-related daily activities is employed for the training, validation, and testing stages. An external experimental test set is constructed, with ten subjects performing the four actions at three different distances and three orientations. The MediaPipe Holistic, Pose, and Hands models [22] are used on RGB images to extract the relevant joint coordinates, which are used as input feature vectors for the ML model.

With the aim of providing a continuous monitoring system, an inference time study was conducted on the implemented Raspberry Pi 4B edge node for each of the MediaPipe models with different degrees of architectural complexity. All the development phases of the model were performed on a personal computer, but the final framework of MediaPipe features and ML model was tested to run directly on the Raspberry Pi 4B. The final goal was to achieve a system that records and processes the data locally, meaning that all the required computing power is on the edge. This paper proposes an approach based on established state-of-the-art machine learning models. The innovative aspect is in the way in which the feature extractor is used, in its evaluation, and in the implementation on the edge, where we show the performance. From a methodological perspective, we investigated multiple options as a classifier, making for a total of 29 typical models, along with a set of different feature vectors and preprocessing steps. Moreover, training the models on the most complex MediaPipe feature extractor and then testing their performance both in terms of latency and F1-score while varying the complexity of the feature extractor is an evaluation that, to the best of our knowledge, has yet to be provided in the literature. This kind of tuning in regard to model complexity is necessary when using platforms with small computational power such as the Raspberry Pi.

An analysis of the best feature vector and the best ML model was performed on a public dataset with a 5-fold cross-validation. The results showed that the most complex MediaPipe architectures for extracting pose and hand keypoints perform the best when operating at one frame per second. A Light Gradient Boosting Machine classifier taking the pose and hand keypoints as input can achieve a weighted F1-score of more than 94% with at least 7 s of observation time and about 5 ms of inference time. The classifier works best when the subject is facing frontally towards the camera, with the performance in this case jumping to almost 97% by weighted F1-score. The model performs even better when the subject is at a distance of around 70 cm from the camera, with the weighted F1-score exceeding 97%.

The rest of the paper is organized as follows. Section 2 presents the employed datasets and hardware platforms. Section 3 describes the process of feature extraction using MediaPipe models, including a description of the preprocessing steps and the list of analysed feature sets. Section 4 describes the method for selecting the best feature set and best ML model, followed by a subsection on performance metrics. The results are shown in Section 5, where the latency of each of the MediaPipe models is evaluated on the Raspberry platform, the best ML models are highlighted, and the cross-validation metrics are reported. All performance metrics are reported on both the internal Kinetics-700 test set and on the experimental test set. Performance is stratified according to subject distance and orientation, and an ablation study is performed. Finally, Section 8 concludes the paper.

## 2. Materials

We employed two datasets for the development of the classification algorithm. The Kinetics-700 public dataset was used for training, internal validation, and testing. A second dataset was constructed within the laboratory and subsequently employed as an external testing set.

### 2.1. Public Dataset and Data Cleaning

For the development of the model, we employed a subset of the Kinetics-700 dataset [25] relating to actions performed by a person in the context of self-hygiene. The dataset contained videos people of brushing their teeth, fixing their hair, or washing their face. We excluded some of the videos in each class based on the following criteria:

- Videos involving multiple people, animals, toys, or other objects.
- Videos containing mirror reflections.
- Videos with the subject recorded from the back.
- Videos with the hands and face completely invisible or outside the video frames; occlusion of hands and face due to normal movement is acceptable.
- Videos with excessive camera movement.
- Videos with excessive corruption or editing.
- Videos of children performing the action incorrectly.

The remaining videos were edited to remove frames where the action was not being performed; the frames removed in this way were saved as a fourth class, denoted ‘no action’. The use of the Kinetics-700 dataset [25] to develop the model enabled us to identify the typical motion patterns of people performing the actions in conditions with different backgrounds, light conditions, and light sources and with videos taken at different resolutions.

After skimming, the classification model was developed based on 91 videos for the teeth brushing class, 162 for the fixing hair class, 242 for the washing or scrubbing face class, and 221 for the no action class.

We divided the dataset into a development set and a testing set with the ratio of 90% and 10%. The development set was then further divided into training and validation sets to choose the best feature extractor, the best feature set, and the best performing ML model. The test set was employed to evaluate the performance of the best model on new and unseen data.

### 2.2. Experimental Dataset

**Figure 1:**
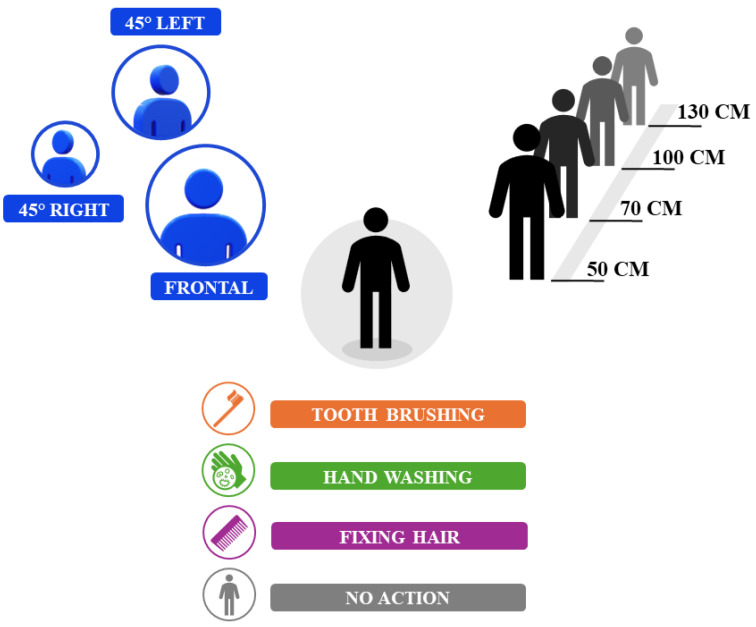
Scheme of the experimental setup with different orientations, distances, and performed actions.

Data were collected from ten volunteers performing the four actions while recording 300 frames at 30 frames per second (fps) on an Intel D455 depth camera at three different distances with the maximum possible resolution. Figure 1 shows a schematic representation of the evaluated trials.

Five subjects were recorded at distances of 50 cm, 70 cm, and 100 cm and the remaining subjects at 70 cm, 100 cm, and 130 cm. For all tested distances, the subjects performed the action with three different body orientations: frontally to the camera, rotated 45° to the left with respect to the camera, and rotated 45° to the right with respect to the camera, as shown in Figure 1. All four considered actions were performed by the subject in all tested conditions. The subjects were allowed to move according to their typical behavioral patterns. We set the distance with a tape measure and checked it with the depth values from the Intel RealSense.

The experimental dataset was not used at any stage in the development of the models, as it was intended as an external test set for evaluating the performance of the final best model across different subject orientations and distances.

### 2.3. The Hardware Platforms

**Figure 2:**
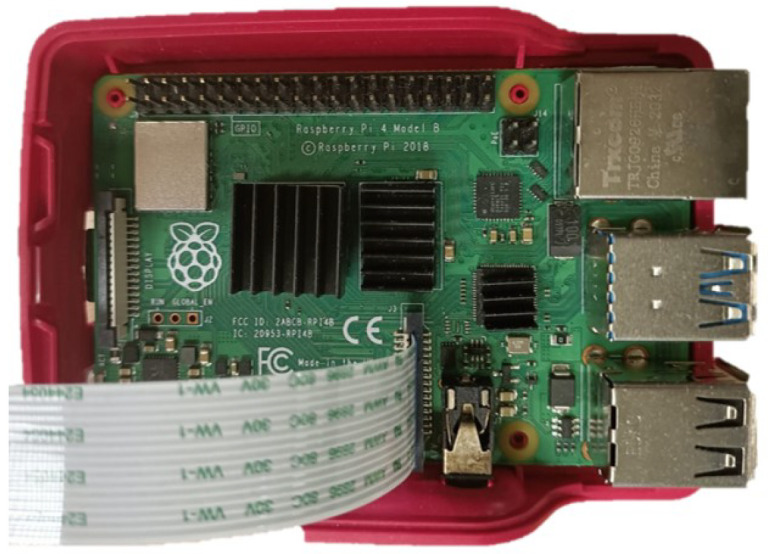
Raspberry Pi4 model B.

A Raspberry Pi 4 model B [26] (Figure 2) was used to carry out the project.

The Raspberry Pi 4B has two micro-HDMI outputs and requires micro-HDMI to HDMI cables or adapters. Algorithms for processing on the board were implemented with Python. The development, validation, and testing of the model were first performed on a personal computer and then adapted to the Raspberry Pi 4B platform, where we evaluated the performance in terms of latency. The limited computational resources of the hardware are shown in Table 2. For recording the experimental dataset, we employed an Intel RealSense D455 camera and a personal computer. Even though we recorded both RGB values and depth values, all processing was performed on the RGB channels, as the intended final hardware was a Raspberry Pi recording with a Pi Camera.

## 3. Feature Extraction

**Figure 3:**
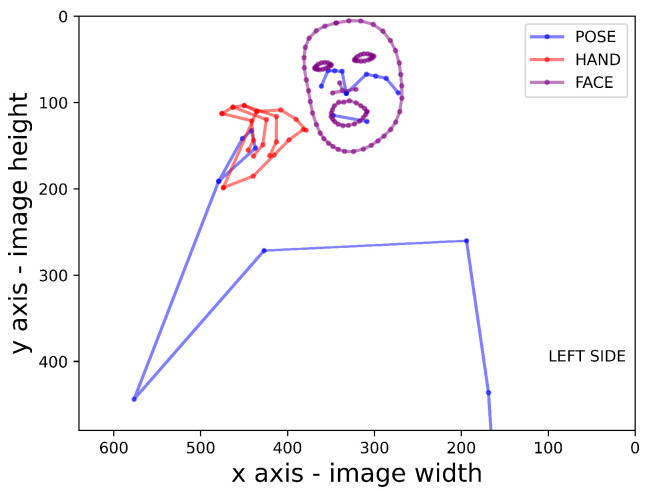
Example of keypoint extraction.

The first step of the proposed approach involves the use of the MediaPipe framework. MediaPipe allows the coordinates of keypoints to be extracted from the pose, hands, and/or the face of the subject performing the action. Figure 3 shows an example scheme of the possible keypoints extracted from a subject. The pose model permits the extraction of 33 three-dimensional points, with a fourth dimension provided by the visibility. The third dimension is discarded in the pose model, as the model specifications suggest that it is prone to error [27]. We only use the first 23 keypoints, which relate to the upper body. The hand model provides 21 three-dimensional coordinates, while the face model can provide up to 478 three-dimensional points describing a face mesh. For the face keypoints, we consider only the face silhouette, the eye contour, and the lip contour in order to simplify the training stage and reduce the dimension of the input features. By default, the vertical coordinates are normalised to the image height in pixels and the horizontal coordinates are normalised to the image width in pixels. The origin point is set to the upper left corner of the frame. The coordinates of the third dimension for the hands are provided in relation to the wrist position, while the coordinates of the third dimension for the face keypoints describe their depth with respect to the center of the head.

The features extracted for each frame are then flattened into a feature vector, which is sent as input to the final model. We collect and combine these features to find the best possible feature extractor in terms of performance and latency metrics on the Raspberry Pi board. We consider two sets of features: one related to the use of a holistic model, where the face, pose, and hands are all possible features for tuning, and a second relating to the simultaneous use of pose and hand models with the keypoints of the pose and hands. As a preprocessing step, we set the origin of the X-axis and Y-axis of the keypoints with respect to the coordinates of the nose provided by the pose model; we further normalise all X–Y coordinates of the keypoints using the shoulder-to-shoulder distance.

The models for the pose (P) and hands (HA) have three and two levels of architectural complexity, respectively. Higher complexity corresponds to a higher number of network parameters, higher accuracy of keypoint estimation, and higher computation time; therefore, it is used for tuning in conditions of low computational resources. The pose model, also called BlazePose [28], comes in three different sizes and capacities. BlazePose “Lite”, which we call the pose model with complexity 0 or “P0”, is the lightest available model in terms of its number of parameters and latency. Next, BlazePose “Full” corresponds to the pose model with complexity 1 or “P1”. Finally, the model with the largest complexity is BlazePose “Heavy” or “P2”. For the model of the hands, “Lite” and “Full” models are available, respectively corresponding to 1 and 1.98 million parameters [29]. The “Lite” model is the model with complexity 0 or “HA0”, while the full model “HA1” has a complexity of 1. Another model is the Holistic MediaPipe model (H), which allows the extraction of pose, face, and hand data, with slightly higher efficiency in face and hand extraction. The Holistic model exploits the pose predictions from the same BlazePose models and then crops the region of the image where the hands and face should be to apply a spatial transformer for keypoint regression, achieving a reduction in inference time of about 90% compared to the corresponding hand and face models. For this reason, the holistic models have three levels of complexity corresponding to the aforementioned three levels of pose complexity used as the backbone for the hands and face keypoints predictions.

A list of the feature vector, the corresponding MediaPipe feature extractor, and the added preprocessing steps is reported in Table 3; from now on, we call these the feature sets and code them with the reported letters. During training and validation of the machine learning models, we decided to employ only the most complex feature extractors. The ones employed were the most complex Holistic model “H2” and the combination of the most complex pose and hands models, “P2HA1”. In this way, we trained the models on the best keypoint coordinates. For this reason, Table 3 reports only these two feature extractors. However, the features that we extracted in terms of the keypoint coordinates (columns 3, 4, and 5 in Table 3) and the preprocessing steps (columns 6 and 7 in Table 3) were the same in training, validation, and testing. Instead, in the testing phase we wanted to understand whether the use of models with lower complexity could provide similar performance to the highest complexity models. While the models with lower complexity have the same input and output as the higher complexity models, they can provide smaller latency due to the smaller number of performed operations, though at the cost of higher estimation errors in the keypoint coordinates. Therefore, evaluating how the performance changes with varying MediaPipe model complexity is valuable for contexts with limited computational power.

## 4. Selection of the Best Feature Set and ML Model

The modified Kinetics-700 dataset was divided into a development set and testing set at a ratio of 90% to 10%, respectively. We initially trained 29 common ML models with the LazyPredict Python library on 50% of the development set, reporting the results on the remaining 50% of the examples to choose the five best performing models based on the weighted F1-score for further evaluation. Additionally, we repeated the training procedure for the 29 available ML models for all the feature set described in Table 3. This first step is needed to reduce the group of possible ML models to a smaller number that achieve the best performance on unseen data. The feature vector was processed by default by substituting Not a Number (NaN) values with the mean of the vector and then performing a standard scaling procedure by removing the mean and dividing by the standard deviation of the training samples. The treatment of NaN values was investigated in detail after selecting the five best performing models by changing the preprocessing between simple mean substitution (mean), zero substitution (zero), and mean substitution plus standard scaling (scal).

The examples in the development set were first cleaned depending on the visibility of the keypoints. For the training stage validation stages, we excluded all frames where keypoints did not appear or where more than 50% of the keypoints for both the hands and arms did not appear. All subsequent evaluations and tests were carried out in normal working conditions with all the frames included, even those in which the keypoints did not appear due to occlusion.

The selected best five models and the best feature sets were trained with 5-fold cross-validation on the entire development. The best model was selected for the successive testing phase. The best model was then used to classify the single frames. It was also evaluated on multiple consecutive frames while providing the most frequent label in the considered frames as output.

### Performance and Latency Metrics

We measured the classification performance on the validation set and test set using typical metrics, i.e., weighted precision, weighted recall, weighted F1-score, per-class precision, per-class recall, and per-class F1-score. Precision, recall and F1-score for a classification task are defined as follows

(1) $$Recall=\frac{TP}{TP+FN},$$

(2) $$Precision=\frac{TP}{TP+FP},$$

(3) $$F1score=2\frac{Precision\times Recall}{Precision+Recall},$$

where *TP* stands for “True Positives”, *FN* for “False Negatives”, and *FP* for “False Positives”. Each of these metrics was computed for each class. In the per-class metrics, one class is considered the positive prediction class and the other classes are clustered in the negative prediction class. In this way, it is possible to view any N-class classification as N binary classification tasks for which the precision, recall, and F1-score can be evaluated. The weighted metrics are obtained by a weighted average of the per-class metrics, with the weights provided by the number of examples in each class. The macro averages are instead unweighted averages of the per-class metrics. If the number of examples is the same for each class, then the macro and weighted metrics will be the same.

The performance evaluation was conducted both on single frames, referred to as per-frame metrics, and consecutive frames, where the most frequent class between frames is reported as the classification output. The best model was evaluated on different observation windows ranging from 1 s to 10 s.

For the evaluation of consecutive frames, we sampled the videos in the test sets with sampling rates of 30 fps, 3 fps, 2 fps, and 1 fps in order to better understand the robustness in conditions of reduced fps, which is typical for the Raspberry Pi 4.

To understand the possibility of processing data directly on the board without the need for cloud computing, we further evaluated the latency of the classification task, as described in the next section.

## 5. Results and Discussion

In this section, we explore in detail the results achieved by the different ML models considered based on evaluation with a hold-out validation set and cross-validation.

### 5.1. Latency and FPS for Each Feature Extractor on the Raspberry Pi 4

**Figure 4:**
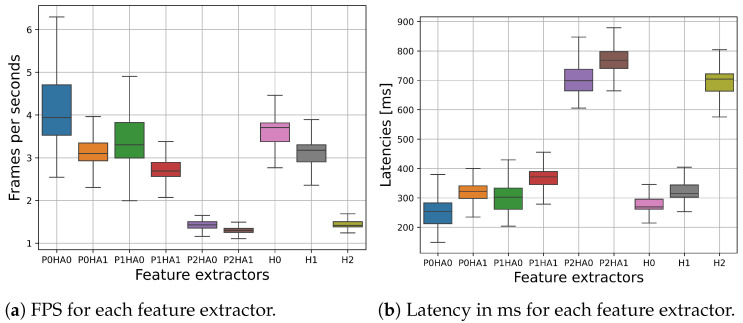
Latency per frame and relative fps for each MediaPipe feature extractor.

The latency of the Holistic MediaPipe model (H) with the three available model complexities (H0, H1, H2) was evaluated on the public dataset, making for a total of 720 frames taken from a random 10% of the videos in the development set. Moreover, we evaluated the latency when using only the pose model (P) with the three available model complexities (P0, P1, P2) in combination with the hands model (HA0, HA1). All of the latency values are reported in fps in Figure 4a and in milliseconds in Figure 4b.

We observe that the median fps results for P0HA0, P0HA1, P1HA0, H0, and H1 are all between 3 and 4, corresponding to a latency per frame of around 300 ms; P1HA1 has median fps between 2 and 3, with latencies per frame between 300 and 400 ms. The most complex models using the highest complexity for the pose models, namely, P2HA0, P2HA1, and H2, work best between 1 and 2 fps, which corresponds to a latency per frame between 700 and 800 ms. In general, increasing the complexity of the architectures used for the pose and hands models increases the inference time for prediction of the keypoints. The highest jump in latency is seen for the feature extractor using the most complex pose models. As previously mentioned, the Holistic 2 (H2) model is slightly more efficient compared to its P2HA1 counterpart.

### 5.2. Selection of the Best ML Model and Best Feature Set

**Figure 5:**
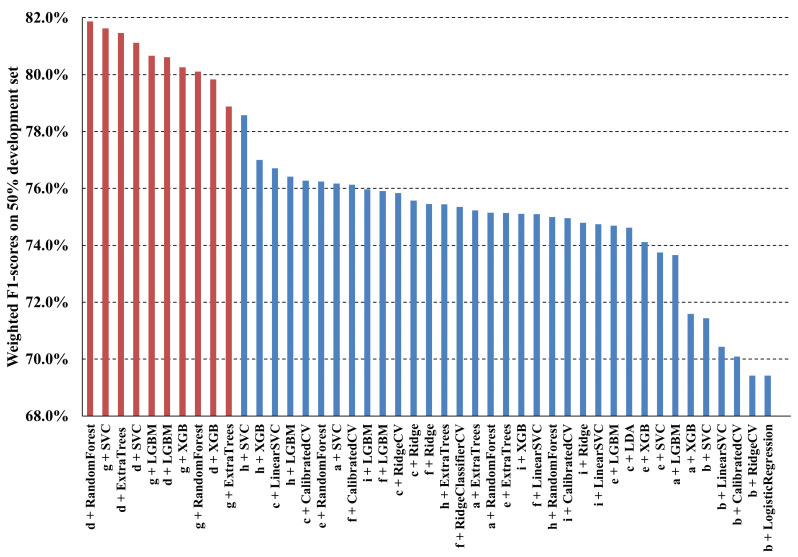
Weighted F1-scores of the five best performing models for each feature set.

In Figure 5, we order the weighted F1-scores computed on a random 50% sample of the development set for the top five best performing models for each feature set listed in Table 3. The top ten F1-scores are composed of the top five performing models for feature sets (d) and (g). These two feature sets were extracted with the P2HA1 feature extractor, and both consist of two-dimensional pose and three-dimensional hand models with respect to the nose keypoints as the origin of the XY plane. The only difference is that normalisation based on shoulder distance is applied to the X and Y coordinates in feature set (g). It can also be noticed that the five best resulting ML models are the same, i.e., Random Forest, Support Vector Machine (SVC), Extra Trees Classifier, Light Gradient Boosting Machine (LGBM) Classifier, and Extreme Gradient Boosting (XGB) Classifier.

In order to clearly obtain the best model and the best feature set based on repeated evaluations, we exploited a 5-fold cross-validation on the development set. Table 4 reports the median and interquartile ranges of the weighted per-frame F1-scores for the five aforementioned models, with the two feature sets d and g used as input. We also evaluated three different strategies for substituting the NaN values in the input feature vector: mean value substitution (*mean*), zero substitution (*zero*), and mean values substitution with standard scaling (*scal*). The latter is the default used by LazyPredict, which was the library used to select the best performing ML models. It is possible to observe that LGBM, with a median of 78.38% (interquartile range 77.39–80.77%), performs slightly better than the other models in all the experiments; feature set (g) with the *mean* substitution strategy provides the best performance. Furthermore, it can be seen that feature set (g) leads to slightly better performance than feature set (d) for the same model and input strategy.

The LGBM classifier was trained on all the development sets; from now on, this configuration is used as the best model in the tests performed on the internal Kinetics-700 test set and the external experimental test set. The configuration parameters of the model were the default ones; thus, the model takes as input the feature set (g) with a dimension of 1 × 170 comprising for the pose coordinates (22 x–y values) and hand coordinates (21 x–y–z values for two hands).

### 5.3. General Metrics Evaluation on the Test Sets

We tested the best model on a 10% hold-out test set from the Kinetics-700 dataset. The test set was composed of examples not seen by the model in any of the development stages. The model was trained to provide a classification for each frame; therefore, Table 5 shows the evaluation metrics for predictions performed on each individual frame. The weighted precision, recall, and F1-score are all greater than 81%, at 81.91%, 81.64%, and 81.68%, respectively. Moreover, we computed the metrics with the observation window increased to all the frames of each video. The model always works by providing a prediction for each frame; we selected the most frequent prediction for all the frames included in the test video. The metrics for all the frames can be seen in Table 6. A clear increase of more than 10% in all the employed metrics can be observed when enlarging the observation window. The main issue is that the length of each video is different; thus, in some certain the effect can be more pronounced than in others. For this reason, we also used an external test set where all the examples contained the same number of frames.

Table 7 and Table 8 show the results obtained on the experimental dataset. It can be seen that the results are more optimistic than the previous ones due to the more controlled conditions in terms of distance and subject orientation in the experimental setup as well as to the larger number of frames.

Given the same number of frames and examples, the macro and weighted metrics are the same. The per-frame weighted metrics are 88.16%, whereas with all frames the metrics increase to 96.39%, representing a reduction of false positives and false negatives in the model’s prediction. This result is expected, as increasing the observation time mitigates some of the error. Certain classes are more positively affected than others, such as “washing face” class with a 10% increase in weighted F1-score and the “fixing hair” class with almost a 12% jump in weighted F1-score. The “no action” class sees less than a 5% increase in F1-score, and “brushing teeth” increases by little more than 7.5% in the same metric.

### 5.4. Metrics for Each Feature Extractor

Even though we trained the model on the features extracted by P2HA1, we are also interested in understanding whether the features extracted by lighter models might be sufficient for achieving comparable accuracy. In Table 9, we report the weighted F1-scores for each combination of pose and hand models between the feature extractors for all the frames. It can be observed that the best performance is achieved by the most complex feature extractor, P2HA1, with an F1-score of 96.39% for all frames, followed by P1HA1 and P0HA1, both with 95.83%. The same three feature extractors have the best performance for the “brushing teeth”, “fixing Hair” and “washing face” classes, reaching higher values in Table 9. These three feature extractors share all the same hands features extracted by the HA1 hands model. It can be observed that the HA1 hands model exhibits better performance, followed by HA0 and the holistic features. This can be attributed to the fact that the hand features are more reliable and accurate when using HA1 than the other alternatives.

For the “washing face” class, the performance seems to be affected by the complexity of the pose model, with increasing performance for increasing complexity in the top performing feature extractors, whereas the pose model does not seem to be determinant for the performance of the other two classes.

For the “no action” class, the top three performing feature extractors are, in ascending order, P2HA1, H2, and P2HA0, with respective F1-scores of 96.09%, 96.17%, and 96.67% on all frames. It can be observed that they share the same pose model complexity and that the hand features seem less relevant in terms of the order of the performance.

### 5.5. Analysis of the Influence of Time Window and FPS on Performance

**Figure 6:**
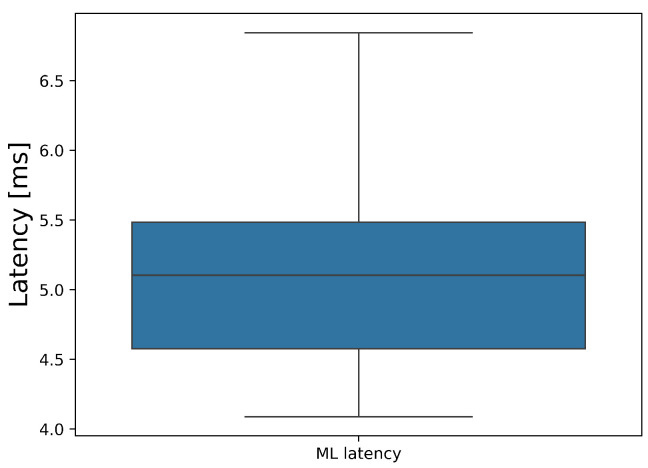
Latency in ms for the chosen machine learning model.

**Figure 7:**
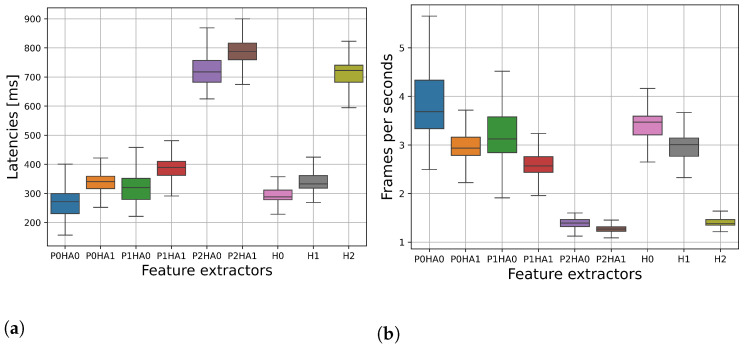
(**a**) Latency per frame and (**b**) relative fps for each MediaPipe feature extractor with the added preprocessing steps and ML model.

The LGBM model has a median latency per frame of 5.10 ms (interquartile range 4.58–5.48 ms), as can be seen in Figure 6. The effect of adding the LGBM model and preprocessing steps on the overall latency and fps of each feature extractor can be seen in Figure 7. The latency and fps were computed as previously described.

**Figure 8:**
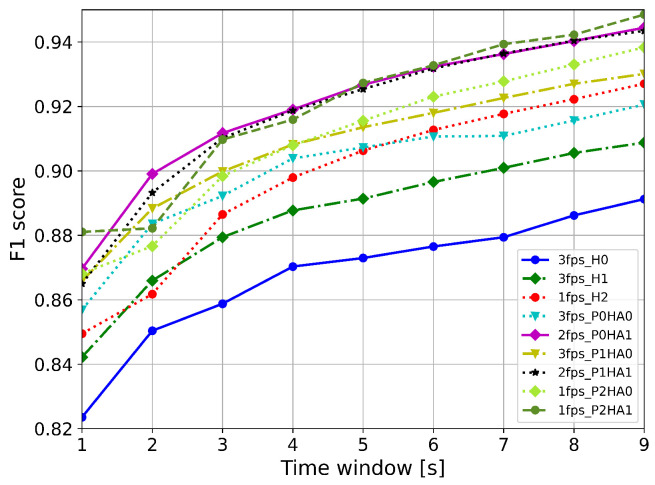
Weighted F1-scores computed on the experimental dataset for different feature extractors. Each feature extractor was tested under the same fps conditions computed for the Raspberry Pi.

Figure 8 reports the F1-scores computed for each feature extractor along with their relative fps, rounding the median fps obtained on the Raspberry Pi 4 down to the nearest integer.

**Figure 9:**
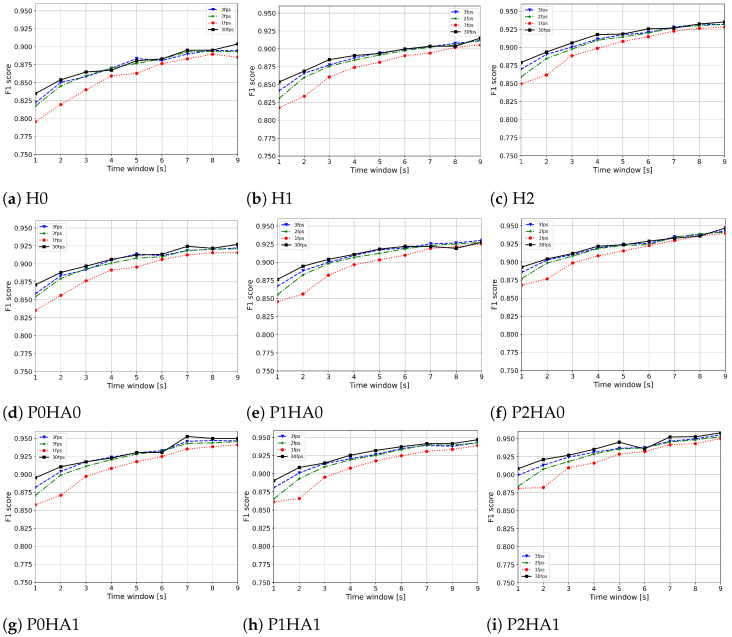
Weighted F1-scores computed on the experimental dataset for all possible features extractors with different fps and increasing the observation window in steps of 1 s.

We used these fps values to downsample the videos in the experimental dataset. We report the weighted F1-scores when increasing the observation window from 1 s to 9 s with a step of 1 s. It can be observed that the P2HA1 feature extractor, although it is the most complex and has the lowest median fps, obtains the best weighted F1-score with an observation window of at least 6 s. From 7 s, the model achieves weighted F1-scores of at least 94%. For observation windows of less than 6 s, the P0HA1 feature extractor working at 2 fps achieves the best overall score, followed by P1HA1 working at 2 fps. It can be observed that the top three performing feature extractors all use HA1 for the hand keypoints, which is the most complex architecture for this extraction. Figure 9 shows the trends in weighted F1-score for all possible MediaPipe feature extractors for increasing observation windows of 1 s to 9 s with a step of 1 s, labelled to indicate 30 fps, 3 fps, 2 fps, or 1 fps. As expected, lower fps values correspond to lower scores; however, the difference becomes less evident as the observation time increases.

### 5.6. Comparison with the State of the Art

In a previous work [24], we proposed an LSTM classifier to address the same four actions, reaching up to 92.8% average accuracy in a 10 s window. To train a classifier that could operate effectively within the limited capabilities of a Raspberry Pi, we chose to develop a simpler machine learning-based system. From the scientific literature, LSTM networks have proven to be effective in recognising dynamic actions related to hygiene [9,20]. However, their main limitation is in the reported latency. For a Raspberry Pi 4, LSTM is reported to have inference time of 500 ms [20], whereas on a CPU it can have a latency of 120 ms [9]. The system proposed in this work reaches similar performance, with a weighted F1-score of at least 94% in a window of seven seconds or more, while working with a latency of around 5 ms. In this way, we achieve the goal of a lightweight alternative to the previously validated deep learning approach.

## 6. Robustness of the Proposed Approach

In this section, we analyze the robustness of the proposed approach under different environmental conditions along with its dependency on the different features employed.

### 6.1. Robustness to Subject Orientation

**Figure 10:**
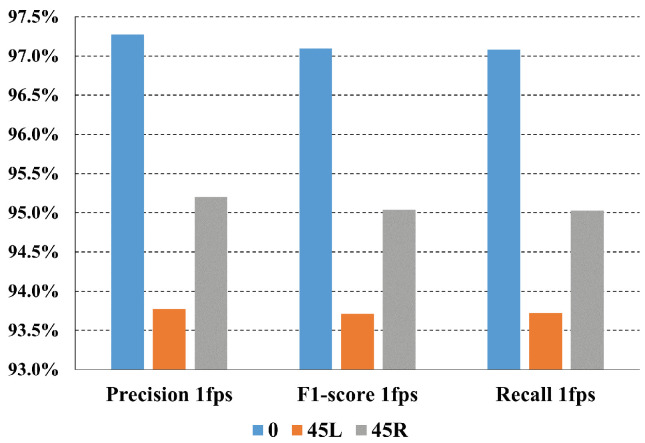
Weighted metrics for each tested orientation.

The experimental dataset is organised in three possible subject orientations, i.e., frontal, rotated 45° to the right (45R), and rotated 45° to the left (45L). The metrics relating to each these three orientations are shown in Figure 10.

The weighted precision, recall, and F1-score were computed for the best feature extractor, P2HA1.As might be expected, the 0° orientation, corresponding to a subject directly facing the camera, achieves the best performance, reaching more than 97% for all metrics, followed by 45° right, where the metrics slightly exceeds 95%, and finally by 45° left, where all the metrics are under 94%. These differences could be due to the fact that the model makes inferences mainly thanks to the X–Y coordinates of the pose and hands of each subject. This could account for the better performance on subjects with a 0° orientation compared with a slanted orientation, as the motion of the actions evolves mainly in the X–Y plane.

### 6.2. Robustness to Subject Distance

**Figure 11:**
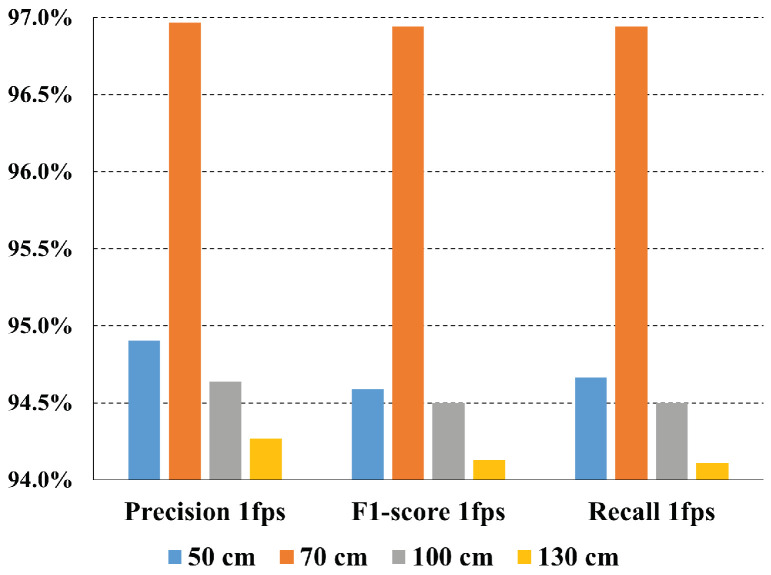
Weighted metrics for each tested distance.

The experimental dataset contains subjects at four possible distances, i.e., 50 cm, 70 cm, 100 cm, and 130 cm. Therefore, we stratified the metrics for all the models between these distances to understand whether they have a significant impact on model performance. Figure 11 shows the weighted metrics computed for each distance, using the best P2HA1 feature extractor and downsampling the videos at 1 fps, the normal working conditions for the chosen extractor.

Interestingly, the closest distance is not the one that provides the best results. In fact, 70 cm is the distance that achieves the best overall performance in terms of weighted precision, recall, and F1-score, reaching almost 97%. This means that 70 cm is the distance at which the best model has the least number of false positive and false negative predictions. This is followed by 50 cm, where the model slightly exceeds 94.5% in all metrics, then 100 cm, where it reaches 94.5% in all metrics, and lastly by 130 cm, where the performance drops by about 0.5 percentage points.

### 6.3. Feature Ablation Study

The complexity of the pose model and hand model affects the performance. Consequently, the trained model was provided with the same input vector, with a group of the features set to NaN in order to ascertain their impact on the overall performance. The experiments included the ablation of:

- All pose keypoints (*nopose*).
- All hand keypoints obtained from the hands model (*nohands*).
- The X–Y coordinates of the hands obtained from the hands model (*nohandsXY*).
- The Z coordinates of hands obtained from hands model (*nohandsZ*).
- The keypoints of the pose face corresponding to the first ten X–Y coordinates (*noposeface*).
- The keypoints of the pose arms corresponding to the X–Y pose coordinates of the shoulders, elbows, and wrists (*noposearms*).
- The keypoints of the pose hands corresponding to the X–Y pose coordinates of the thumbs and fingers (*noposehands*).

**Figure 12:**
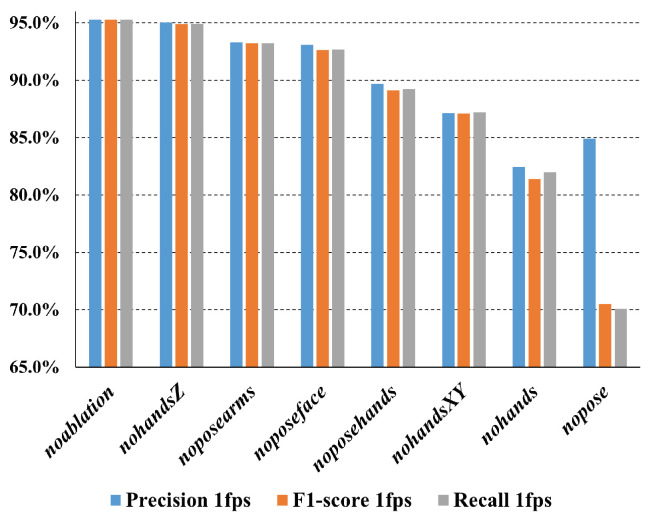
Weighted F1-scores for the ablation experiments.

Table 10 reports the metrics obtained with the ablation experiments for the normal working conditions with the P2HA1 feature extractor predicting a 10 s video at 1 fps. Figure 12 shows the weighted F1-scores in descending order and how each ablation experiment causes corresponding performance degradation in the action classification.

Figure 12 illustrates the relevance of certain features for classification when comparing the ablation study with the case of no ablation. Table 10 demonstrates how each feature is relevant for a specific class. The Z-coordinate of the hands appears to have a relatively minor impact on the performance (between 0.3% and 0.4%), followed by the absence of the pose arms and pose face (around 2%). The first large negative jump is seen with the absence of the pose hands, which negatively affects the recall for all actions with the exception of “washing face“. A clear decline in performance is also evident in the absence of the X–Y hand coordinates, with a reduction of approximately 8% observed across all metrics.

Finally, it can be observed that the pose coordinates are the most relevant features for making accurate predictions. It can be observed that in the absence of pose information, the precision of the “no action” class is almost halved to 46.50% in favour of the other classes. Conversely, the recall of “brushing teeth” and “fixing hair” is increased; in the condition where no information about the pose is available, the model tends to predict the “no action” class, as it does not see a body, only the keypoints of the hands. This indicates that the model requires information about the pose to predict the “brushing teeth” and “fixing hair” classes, whereas the “washing face” class is less affected by the absence of pose coordinates. In the case of the “washing face” class, the model primarily utilises the positions of the hands in relation to the nose coordinates for prediction.

## 7. Issues and Future Directions

There are several issues that could be addressed in future work, which we outline below. In this paper, we have proposed an extensive analysis with a set of machine learning algorithms for the classification of four hygiene related actions. The rationale behind the choice of this set of simple actions was to predict whether or not basic self-care is being carried out by the monitored subject. However, this closed set of activities could be enlarged to cover other activities of daily living with fewer intra-class differences, although this would make the classification task harder. Moreover, we have focused only on the analysis of the RGB channels, which could produce privacy concerns. A possible next step would be to exploit depth data to perform similar classification of activities in a domain that is less informative, thereby helping to protect the private life of the monitored subject. Another concern is the potential overfitting of the supervised model. While overfitting is a challenging issue to fully avoid, we have addressed it by training a simple model on an external dataset containing numerous examples from the daily lives of different people, all of which include various lighting conditions and resolutions. Additionally, using the MediaPipe feature extractor serves as a form of dimensionality reduction and pattern extraction, simplifying the training and reducing the overall complexity of the required model. Finally, we performed an evaluation of the initial feature vector provided as input to the model, based on which we removed the facial feature, further simplifying the model to prevent overfitting. However, the final experimental test set is constrained by the size of the population. The tested activities all occurred in a controlled laboratory environment, and as such may differ slightly from typical subject behaviors. Additionally, the same actions may vary when performed by people of different ages. Future developments could explore the system’s performance with a sample size that includes only elderly people.

## 8. Conclusions

This paper has proposed an ML-based approach that aims to provide constant monitoring of self-hygiene activities, the decline of which is often related to loss of autonomy and the onset of frailty status in elderly people. Moreover, we implemented an edge node on a Raspberry Pi4, taking into account all the related constraints in computing and storage capacity. RGB frames extracted from videos were initially processed using MediaPipe algorithms to identify the keypoints of the pose, hands, and if necessary the face. Different ML models and feature extractors were tested on the Kinetics dataset, then applied to an experimental dataset which included four actions with different subject orientations and distances. The final configuration of the feature extractor and model achieved a weighted F1-score of over 94% under 1 fps conditions, along with an observation time of 7 s or more. The proposed model performs optimally in conditions where the subject is facing directly towards the camera, achieving more than 97% weighted precision, recall, and F1-score with a 10 s observation window at 1 fps. Furthermore, it performs optimally when the subject is positioned at 70 cm from the camera, reaching almost 97% weighted precision, recall, and F1-score with a 10 s observation window at 1 fps. Finally, an ablation study on the different features demonstrated that the pose keypoints are the most relevant features, while the Z-coordinate has a negligible impact on the results.

## Figures and Tables

**Table 1:** Differences with the state of the art.

Ref.	Hygiene-Related Activities	MediaPipe	Raspberry Pi4	Edge Computing
[11]	✓	✓	✗	✗
[23]	✓	✗	✗	✗
[13]	✓	✓	✗	✗
[14]	✓	✗	✗	✗
[15]	✓	✗	✗	✗
[9]	✓	✗	✗	✗
[10]	✓	✗	✗	✗
[19]	✗	✓	✓	✓
[20]	✗	✓	✓	✓
[21]	✗	✓	✗	✗
[24]	✓	✓	✗	✗
This paper	✓	✓	✓	✓

**Table 2:** Raspberry Pi 4 model B technical specifications.

Specifications	
Processor	quad-core Cortex-A72 (ARM v8) 64-bit
Clock speed	1.5 GHz
Memory	8 GB

**Table 3:** List of feature extractors and feature sets employed in the development phase. All feature sets were evaluated with 29 common ML models on a 50% sample of the development set. For each feature set, the table shows the respective feature extractor (MediaPipe model), extracted keypoints, and the preprocessing steps.

Feature Set	Feature Extractor	Keypoints			Pre-Processing	
		Pose	Hands	Face	Origin Nose	Norm by Shoulder Distance
(a)	P2HA1	✓	✓	✗	✗	✗
(b)	H2	✓	✓	✗	✗	✗
(c)	H2	✓	✓	✓	✗	✗
(d)	P2HA1	✓	✓	✗	✓	✗
(e)	H2	✓	✓	✗	✓	✗
(f)	H2	✓	✓	✓	✓	✗
(g)	P2HA1	✓	✓	✗	✓	✓
(h)	H2	✓	✓	✗	✓	✓
(i)	H2	✓	✓	✓	✓	✓

**Table 4:** Median weighted per-frame F1-scores for the top five performing models on the development set obtained with 5-fold cross-validation. We evaluated two different feature sets (d and g) and three different substitutions of NaN values (mean, scal, zero).

	Mean + (d)	Mean + (g)	Scal + (d)	Scal + (g)	Zero + (d)	Zero + (g)
SVC	76.07 (74.67–78.3)	76,65 (74.27–78.45)	**77.28 (75.83–79.15)**	77.83 (75.07–79.1)	76.08 (74.47–78.27)	76.44 (74.85–78.6)
LGBM	**77.01 (74.15–78.81)**	**78.38 (77.39–80.77)**	76.72 (74.34–78.5)	**78.22 (77.22–80.79)**	**76.96 (74.73–78.69)**	**78.05 (77.45–81.02)**
XGB	76.13 (73.87–77.82)	77.56 (75.91–79.82)	76.15 (73.44–77.66)	77.9 (75.81–79.38)	75.97 (74.02–78.31)	77.98 (76–80.05)
RandomForest	76.59 (73.55–77.87)	77.54 (75.03–78.38)	76.34 (73.9–77.98)	77.82 (75.17–78.56)	76.66 (73.46–77.37)	77.65 (75.39–78.49)
ExtraTrees	76.11 (72.58–77.82)	76.53 (72.61–78.46)	76.61 (72.44–77.57)	76.84 (72.74–78.77)	76.11 (72.7–77.87)	76.78 (73.09–78.53)

**Table 5:** Per-frame metrics on the Kinetics test set.

Classes	Precision	F1 Score	Recall
brushing teeth	0.8219	0.7472	0.6850
fixing hair	0.8661	0.8497	0.8340
no action	0.7569	0.8016	0.8520
washing face	0.8236	0.8257	0.8277
**Metrics averages**			
weighted	0.8191	0.8168	0.8164
macro	0.8171	0.8061	0.7997

**Table 6:** Metrics for all frames on the Kinetics test set.

Classes	Precision	F1 Score	Recall
brushing teeth	100%	100%	100%
fixing hair	90.00%	94.74%	100%
no action	95.45%	95.45%	95.45%
washing face	95.00%	90.48%	86.36%
**Metrics averages**			
weighted average	94.58%	94.39%	94.44%
macro average	94.44%	94.44%	95.45%

**Table 7:** Per-frame metrics on the experimental test set.

Classes	Precision	F1-Score	Recall
brushing teeth	87.24%	88.02%	88.82%
fixing hair	88.66%	84.75%	81.17%
no action	89.71%	91.86%	94.11%
washing face	87.05%	87.79%	88.55%
**Metrics averages**			
weighted	88.16%	88.16%	88.16%
macro	88.16%	88.16%	88.16%

**Table 8:** Metrics for all frames on the experimental test set.

Classes	Precision	F1-Score	Recall
brushing teeth	95.56%	95.56%	95.56%
fixing hair	95.60%	96.13%	96.67%
no action	96.63%	96.09%	95.56%
washing face	97.78%	97.78%	97.78%
**Metrics averages**			
weighted	96.39%	96.39%	96.39%
macro	96.39%	96.39%	96.39%

**Table 9:** Weighted F1-scores and per-class F1-scores stratified for all feature extractors and computed on all frames of the experimental test set at 30 fps; the top three scores for each metric are highlighted.

	Brushing Teeth	Fixing Hair	No Action	Washing Face	Weighted
H0	87.27%	92.97%	94.18%	90.61%	91.26%
H1	86.59%	91.89%	94.12%	94.57%	91.79%
H2	89.16%	93.05%	**96.17%**	95.65%	93.51%
P0HA0	90.06%	92.74%	95.08%	91.98%	92.46%
P0HA1	**96.13%**	**96.63%**	94.44%	**96.13%**	**95.83%**
P1HA0	91.95%	92.82%	95.56%	94.05%	93.60%
P1HA1	**94.92%**	**95.60%**	96.05%	**96.74%**	**95.83%**
P2HA0	93.02%	93.99%	**96.67%**	95.14%	94.70%
P2HA1	**95.56%**	**96.13%**	**96.09%**	**97.78%**	**96.39%**

**Table 10:** Precision, recall and F1-score for each class in each ablation experiment; cells are colored depending on the percentage values, with a three-value gradient from 60% or lower (red) to 80% (yellow) and 100% (green).

F1-Score	Brushing Teeth	Fixing Hair	No Action	Washing Face
*noablation*	95.05%	94.66%	95.06%	96.35%
*nohands*	73.92%	88.14%	88.37%	75.22%
*nohandsXY*	80.42%	87.97%	95.35%	84.63%
*nohandsZ*	94.43%	93.68%	96.06%	95.36%
*nopose*	68.36%	60.57%	63.26%	89.82%
*noposearms*	94.24%	92.07%	92.19%	94.46%
*noposeface*	90.36%	92.67%	93.02%	94.49%
*noposehands*	86.99%	91.88%	84.35%	93.24%
**Precision**	Brushing teeth	Fixing Hair	No action	Washing Face
*noablation*	94.15%	94.80%	95.41%	96.79%
*nohands*	87.90%	85.98%	79.16%	76.80%
*nohandsXY*	84.93%	87.36%	93.58%	82.69%
*nohandsZ*	98.79%	93.15%	92.61%	95.57%
*nopose*	100.00%	98.99%	46.50%	94.08%
*noposearms*	93.10%	90.11%	93.26%	96.78%
*noposeface*	98.68%	93.38%	87.80%	92.48%
*noposehands*	82.82%	92.17%	94.70%	89.10%
**Recall**	Brushing teeth	Fixing Hair	No action	Washing Face
*noablation*	95.96%	94.52%	94.70%	95.93%
*nohands*	63.78%	90.41%	100.00%	73.70%
*nohandsXY*	76.37%	88.59%	97.19%	86.67%
*nohandsZ*	90.44%	94.22%	99.78%	95.15%
*nopose*	51.93%	43.63%	98.89%	85.93%
*noposearms*	95.41%	94.11%	91.15%	92.26%
*noposeface*	83.33%	91.96%	98.89%	96.59%
*noposehands*	91.59%	91.59%	76.04%	97.78%

## Data Availability

The data presented in this study are not available in order to maintain the privacy of the subjects involved. The employed scripts will soon be made available on our Github page https://github.com/TLCunivpm/ML-based-Edge-Node-for-People-Frail-Status-Monitoring (accessed on 3 July 2024).

## Abbreviations

The following abbreviations are used in this manuscript:

DL	Deep Learning
fps	Frames Per Second
IoT	Internet of Things
LSTM	Long Short-Term Memory
ML	Machine Learning
NaN	Not a Number
